# Pathology and molecular mechanisms of Schistosoma japonicum-associated liver fibrosis

**DOI:** 10.3389/fcimb.2022.1035765

**Published:** 2022-10-28

**Authors:** Zhilong Liu, Lichen Zhang, Yinming Liang, Liaoxun Lu

**Affiliations:** ^1^ Laboratory of Genetic Regulators in the Immune System, Henan Collaborative Innovation Center of Molecular Diagnosis and Laboratory Medicine, School of Laboratory Medicine, Xinxiang Medical University, Xinxiang, China; ^2^ Henan Key Laboratory of Immunology and Targeted Therapy, Xinxiang Medical University, Xinxiang, China; ^3^ Institute of Psychiatry and Neuroscience, Xinxiang Medical University, Xinxiang, China

**Keywords:** schistosomiasis, liver fibrosis, molecular mechanisms, soluble egg antigen, cytokines

## Abstract

Schistosomiasis has been widely disseminated around the world, and poses a significant threat to human health. Schistosoma eggs and soluble egg antigen (SEA) mediated inflammatory responses promote the formation of egg granulomas and liver fibrosis. With continuous liver injuries and inflammatory stimulation, liver fibrosis can develop into liver cirrhosis and liver cancer. Therefore, anti-fibrotic therapy is crucial to increase the survival rate of patients. However, current research on antifibrotic treatments for schistosomiasis requires further exploration. In the complicated microenvironment of schistosome infections, it is important to understand the mechanism and pathology of schistosomiasis-associated liver fibrosis(SSLF). In this review, we discuss the role of SEA in inhibiting liver fibrosis, describe its mechanism, and comprehensively explore the role of host-derived and schistosome-derived microRNAs (miRNAs) in SSLF. Inflammasomes and cytokines are significant factors in promoting SSLF, and we discuss the mechanisms of some critical inflammatory signals and pro-fibrotic cytokines. Natural killer(NK) cells and Natural killer T(NKT) cells can inhibit SSLF but are rarely described, therefore, we highlight their significance. This summarizes and provides insights into the mechanisms of key molecules involved in SSLF development.

## Introduction

Schistosomiasis is a zoonotic parasitic disease caused by schistosomes and infects more than 200 million people in 78 countries worldwide (Huang et al., 2020 **).** Schistosomiasis is a major threat to human health and economic development, with more than 200,000 deaths and nearly 800 million people exposed to schistosomiasis infections every year (Huang et al., 2020; Chen et al., 2022). There are six subtypes of schistosomes, though only *Schistosoma japonicum*, *Schistosoma mansoni*, and *Schistosoma haematobium* are responsible for human diseases (Anthony et al., 2010). In the lifecycle of schistosomes, the oncomelania is the unique intermediate host of schistosomes. The oncomelania releases mature schistosoma cercariae into the water, which invades the skin and enters the circulatory system of the host (Colley et al., 2014). As schistosome larvae mature, adult schistosomes attach to the inner walls of the hepatic portal and mesenteric veins, with the aid of suction cups in the mouth and abdomen, to avoid being displaced by high-speed blood flow (Wang J. et al., 2020). The liver is the center of energy metabolism, and the presence of schistosomes in the liver and mesenteric veins is likely related to adequate energy supply. Schistosomes are dioecious, the sexual maturation and egg laying of females are completely controlled by males, and the mating of males and females promotes the production of eggs by females (Chen et al., 2022). The average lifetime of schistosomes in human hosts is 3-10 years, though in certain cases can reach 40 years. A single female schistosome can produce more than a million eggs during its entire life cycle (Colley et al., 2014; Chen et al., 2022). The eggs produced by female schistosomes in the mesenteric vein cause an inflammatory response, and an excessive egg burden can damage the intestinal wall (Clerinx and Van Gompel, 2011). Schistosoma eggs migrate from the damaged intestinal wall into the intestine and are excreted in the feces (Carson et al., 2018). Schistosoma eggs can hatch into miracidia in a suitable natural environment, and the miracidia can infect the oncomelania and develop into mother sporocysts and daughter sporocysts in their bodies, and finally release mature cercariae (Sokolow et al., 2018). Mature cercariae reinfect susceptible hosts and perpetuate the schistosome life cycle.

The large number of eggs produced by female schistosomes is extremely harmful to the host, and over 50% of these eggs are deposited in the liver sinusoids (Anthony et al., 2010). Schistosome eggs and SEA mediate the formation of egg granulomas and liver fibrosis, which are the main pathological features of schistosomiasis (Zheng et al., 2020). In the early stages of schistosome infections, the toxins and antigenic substances released by schistosomes trigger a strong T-helper 1 (Th1) type immune response in the host organism, which mainly manifests by increased expression of Th1-type cytokines, such as IFN-γ, TNF-α, and IL-1, IL-2 (Stadecker et al., 2004). The th1-type immune response has a beneficial killing effect on Schistosoma. However, hosts appear to lack the capacity to eliminate schistosomes, which is also related to the specific immune escape mechanism of schistosomes (Trottein et al., 1999). Continuous inflammatory stimulation leading to excessive Th1 response can accelerate host death due to a cytokine storm (Hoffmann et al., 2000). After 4 weeks of Schistosoma japonicum infection, along with the production of schistosome eggs, the host’s immune response transforms into a T-helper 2 **(**Th2**)** type immune response, which is marked by increased secretion of Th2 type cytokines, such as IL4, IL-5, and IL-13 (Stadecker et al., 2004). Th2-type cytokines promote the polarization of macrophages to M2-type macrophages, while M2-type macrophages and Th2 maintain a high level of type II immune response in their microenvironment (Mei et al., 2022). Type II cytokines inhibit excessive activation of the inflammatory response and promote the formation of egg granulomas (Chen et al., 2014). Egg granulomas are formed by a massive number of eosinophils, neutrophils, macrophages, lymphocytes, monocytes, and aggregates around the eggs (Li L. et al., 2017). The formation of egg granulomas enables the isolation of eggs and toxins, which helps facilitate host survival (Chen D. et al., 2013). However, egg granulomas and type II immune responses inhibit host killing and clearance of schistosomes and lead to the formation of severe liver fibrosis (Hoffmann et al., 2000).

SSLF is a characteristic pathological transformation of the liver caused by schistosome infection, and liver fibrosis is an excessive deposition of extracellular matrix**(**ECM**)**, which is characterized by increased production and decreased degradation of ECM (Huang et al., 2014). This results in the transformation of the ECM into a mesenchymal matrix composed of type I and type III collagen (Chen et al., 2019b). Egg granulomas and liver fibrosis occur in the hepatic sinusoids and portal veins causing increased pressure in the hepatic sinusoids, which can enlarge the liver and spleen, portal hypertension, and varices (Wang et al., 2022). The development of SSLF aggravates hepatic circulation disorders, which aggravates portal hypertension and gastroesophageal varices. The bleeding caused by varices in the lateral circulation of the gastro esophagus is the main cause of death in patients with schistosomiasis (Chatterjee et al., 2004). The proliferation and activation of hepatic stellate cells**(**HSCs**)** is a critical factor in the progression of SSLF. Resting HSCs are located in the Disse region of the subendothelial space and their major function is to stock vitamin A and erythropoietin (Carson et al., 2018; Wang et al., 2022). HSCs are activated to myofibroblasts during liver injuries or inflammatory stimulation, which express α-smooth muscle actin **(**α-SMA**)** and overproduce hydroxyproline-containing collagen leading to massive deposition of ECM (Yu et al., 2016; Zhu et al., 2018). Schistosoma infections induce differentiation of HSCs into two functionally distinct cell subtypes, while MHC II^-^ HSCs exhibit a myofibroblast phenotype, and MHC II^+^ HSCs are associated with regulatory T cell development (Zhou et al., 2017). Inhibiting HSC activity or directly killing HSCs is the main goal of treating liver fibrosis while promoting senescence and apoptosis of HSCs could effectively relieve liver fibrosis (Chen et al., 2016).

Schistosome vaccines have been extensively researched and have a positive effect on the prevention of schistosomiasis (McManus et al., 2020). However, the treatment of schistosomiasis patients has always been highly dependent on praziquantel (Huang et al., 2020). Praziquantel can kill mature schistosomes but is not effective in schistosome eggs, and patients who are reinfected with schistosomes in endemic areas will gradually develop a resistance phenotype to praziquantel (Li Y. Q. et al., 2017). Liver fibrosis is reversible in quiescent circumstances; however, continued damage and inflammatory stimulation will transform liver fibrosis into cirrhosis and cancer (Kamdem et al., 2018). Therefore, the study of antifibrotic treatments for schistosomiasis is significant for improving the treatment and prognosis of schistosomiasis patients.

Previous reviews have extensively discussed the host regulators of SSLF and the genetics of human schistosomiasis (Andrade, 2008; Kamdem et al., 2018; Mewamba et al., 2021). However, there is insufficient discussion in SSLF regarding the role of SEA, schistosome-derived miRNAs and critical pro-fibrotic cytokines. This review outlines some important mechanisms of cytokines in SSLF.

## SEA suppresses the proliferation and activation of HSCs

**Figure 1:**
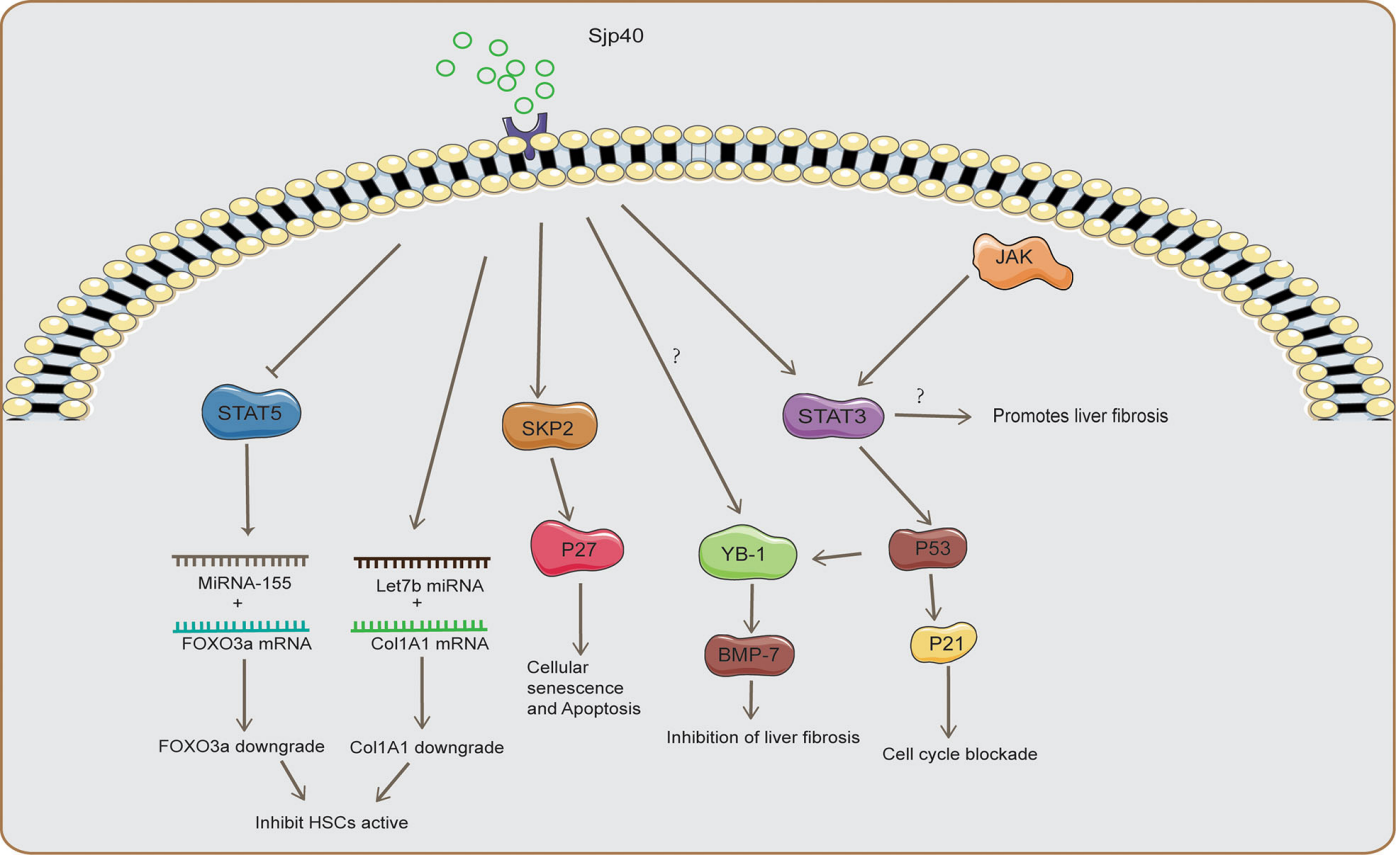
The mechanism of SSLF inhibition by Sjp40. Sjp40 inhibited STAT5 activity to promote MicroRNA-155 expression, which down-regulated FOXO3a to inhibit the proliferation and activation of HSCs and upregulation of Let-7b in HSCs to inhibit type I collagen expression. Sjp40 promotes cellular senescence in HSCs by upregulating the SKP2/P27 signaling pathway. Sjp40 promotes Y-Box protein-1 (YB1) expression to initiate BMP-7 transcription. Sjp40 promoted the P53/P21 signaling to inhibit cell proliferation, and P53 promotes nuclear translocation of YB-1. Inhibition of JAK2/STAT3 signaling could inhibit SSLF.

The activation of HSCs is suppressed by SEA, and is accompanied by decreased levels of a-SMA and Collagen 1A1**(**Col1A1**)** and increased levels of Matrix Metalloproteinase 9 **(**MMP-9**) (** Anthony et al., 2013). This also explains why HSCs tend to be located at the margins of egg granulomas. Collagen deposition in the area close to the eggs is likely detrimental to the survival of the eggs. The composition of SEA considerably varies at different stages of egg development. The first release of Schistosoma japonicum eggs is immature, and the inner envelope of the mature eggs after one week is considered to be the main source of SEA (De Marco Verissimo et al., 2019). SEA is a complex mixture of multiple egg antigens, of which Schistosoma japonicum egg antigen p40**(**Sjp40**)** is one of the components after six weeks of Schistosoma japonicum infection (Zhu et al., 2018; Chen et al., 2019b). Sjp40 is a marker used for early schistosomiasis diagnosis, and sjp40 inhibits the activation and proliferation of HSCs (Zhu et al., 2018). Mechanistically, sjp40 inhibits STAT5 activity to promote MicroRNA-155 expression (**Figure 1**), which down-regulates FOXO3a to inhibit the proliferation and activation of HSCs (Zhu et al., 2018). Lethal-7b (Let-7b) can specifically inhibit type I collagen expression, upregulation of Let-7b in HSCs by Sjp40 inhibits type I collagen expression, and ECM deposition (Sun X. et al., 2021). SKP2 is an important E3 ubiquitin ligase that targets P27 (an inhibitor of cyclin-dependent kinases) and participates in regulating cellular senescence and apoptosis (Wang et al., 2021). Sjp40 promotes cellular senescence in HSCs by upregulating the SKP2/P27 signaling pathway (**Figure 1**), and senescent HSCs show reduced collagen production and increased degradation (Xu et al., 2017). STAT3 is a transcription factor that activates proliferation-related cytokines involved in cell survival and growth, however, STAT3 promotes egg granulomas and liver fibrosis in Schistosoma infections (Zhao et al., 2021). JQ-1 is a small-molecule bromodomain inhibitor that relieves liver fibrosis caused by *Schistosoma japonicum* infections by inhibiting JAK2/STAT3 signaling (Ding et al., 2021). Interestingly, STAT3 signaling also promotes cellular senescence and growth arrest, and phosphorylated-STAT3(p-STAT3) promotes the P53/P21 signaling axis to inhibit cell proliferation (Santarelli et al., 2019). Sjp40 promotes cell cycle arrest in HSCs by activating the STAT3/P53/P21 signaling pathway (Chen et al., 2016). STAT3 is a double-edged sword in schistosome infections and is involved in the activation and senescence of HSCs, which could be related to different sources of signaling with stimulation (Chen et al., 2016; Ding et al., 2021). Bone morphogenic protein (BMP-7) is an antifibrotic protein that inhibits the activity of HSCs, and Sjp40 promotes Y-Box protein-1 (YB1) expression to initiate BMP-7 transcription (Chen B. L. et al., 2013; Chen et al., 2019b). P53 promotes nuclear translocation of YB-1, and STAT3/P53 signaling could regulate HSC activation by regulating nuclear translocation of YB-1 (**Figure 1**). SEA promotes increased production of reactive oxygen species (ROS) and induces apoptosis in HSCs, which could be associated with increased ROS-mediated caspase-1 apoptosis (Kong et al., 2019). However, increased apoptosis of HSCs could play an anti-fibrotic role, but the apoptosis of HSCs aggravates the inflammation and damage caused by schistosome infections. IL-34 promotes M2 macrophage polarization and inhibits NK cell killing of HSCs to promote liver fibrosis; however, SEA inhibits IL-34 expression (Chen et al., 2019a). Peroxisome proliferator-activated receptor γ(PPAR-γ) signaling inhibits HSC differentiation into myofibroblasts, and SEA inhibits HSC activation by increasing the expression of PPAR-γ (Anthony et al., 2010). SEA induces endothelial cell (EC) proliferation and upregulates vascular endothelial growth factor (VEGF) to promote HSC activation (Luo et al., 2017).

The schistosome infection microenvironment is highly complex and diverse, consisting of various parenchymal and non-parenchymal cells (Zhan et al., 2019). Under *in vitro* SEA stimulation conditions, HSCs are quiescent and suppressed, however, in the complex environment *in vivo*, SEA has a bidirectional effect on the development of liver fibrosis (Anthony et al., 2013). HSCs are usually absent from the core area of egg granulomas, and HSC-induced ECM deposition could be detrimental to the survival of the eggs. However, schistosome eggs and SEA construct a type II immune microenvironment, and some critical cytokines have potent pro-fibrotic effects, such as transforming growth factor-beta **(**TGF-β) and IL-13 (Li et al., 2010; Wang S. et al., 2020). This also explains the collagen deposition at the margins of the egg granuloma but the lack of collagen deposition in the core area. Schistosoma eggs and SEA can inhibit the activation and collagen deposition of HSCs, which could be related to the survival of Schistosoma eggs in the host.

## MicroRNA

MiRNAs are a group of highly conserved endogenous non-coding single-stranded RNA molecules that play an important role in regulating gene expression (He et al., 2018a; Wang et al., 2022). miRNAs combine with the 3’UTR region of target mRNAs to inhibit gene expression by promoting mRNA degradation and translation blockage. More than 60% of protein-coding genes contain miRNA target sequences (Huang et al., 2018; Liu et al., 2021a; Sun X. et al., 2021). The miRNA in the liver of schistosome-infected mice originated from the host and schistosome eggs (Wang L. et al., 2020; Xu et al., 2021).

Exosomes of schistosome egg-derived origin transport contents to the receptor cells in the form of secretory vesicles and regulate the functional activity of the receptor cells. These exosomes also contain large amounts of proteins, lipids, and nucleic acids for long-range signal transmission (Wang L. et al., 2020; Wang et al., 2022). Generally, miRNAs promote the degradation of target genes after combining with their mRNAs (Wang J. et al., 2020). Interestingly, *Schistosoma japonicum* egg-derived miRNA-33 upregulates TGF-β receptor expression in HSCs, and miRNA-33 can promote TGF-β receptor expression by enhancing mRNA stability (Wang et al., 2022). Another schistosome egg-derived miRNA-71a targets semaphorin 4D(Sema4D) to play an inhibitory role in liver fibrosis (Wang L. et al., 2020). Wnt/β-catenin signaling is involved in protein construction and modification, and the Wnt/β-catenin signaling pathway can activate HSCs and promote the expression of connective tissue growth factor (CTGF) and TGF-β (Wang Q. et al., 2017). Secreted frizzed-related protein 1 (SFRP1) is an extracellular homologous protein of the Wnt receptor that exerts negative regulation by competing with the wnt receptor to bind Wnt ligands (Zhang H. et al., 2019). Schistosoma-derived miRNA-1 promotes SSLF by targeting SFRP1 in HSCs and upregulating Wnt/β-catenin signaling (Wang Y. et al., 2020). MiRNAs originating from schistosome-infected hosts are also involved in regulating SSLF. FOXO1 is a main downstream effector of the PI3K/AKT signaling pathway, and FOXO1 is involved in regulating the cell cycle, apoptosis, autophagy, and other physiological processes (Xing et al., 2018). MiRNA-182 promotes the activation and proliferation of HSCs by targeting and inhibiting FOXO1 expression (Huang et al., 2018). Let-7b is a member of the Let miRNA family, and Let-7b targets the 3’ UTR of type I collagen mRNA to inhibit collagen deposition in the ECM (Sun X. et al., 2021). Overexpression of Let-7b by recombinant lentivirus transfection dramatically relieves SSLF and suppresses Th1 and Th2 type immune responses (Tang et al., 2017). IL-33 is an important pro-SSLF cytokine. miRNA-203-3p can target and inhibit IL-33 expression, while overexpression of miRNA-203-3p inhibited SSLF by downregulation of IL-33 levels (He et al., 2018b). KH-type splicing regulatory protein (KSRP) is an RNA-binding protein that targets the 3’UTR-rich AU region of TGF-β1 mRNA and promotes degradation by reducing its stability (Sundaram et al., 2013). miRNA-27b increased the mRNA stability of TGF-β by inhibiting KSRP expression and miRNA-27b knockdown could significantly inhibit SSLF (Wang S. et al., 2020). Vitamin D receptor (VDR) signaling promotes the dissociation of the Smad proteome from DNA to inhibit liver fibrosis (Ding et al., 2013). miRNA-351 inhibits VDR signaling and reduces the inhibitory effect of VDR signaling on the Samd proteasome (He et al., 2018a). MiRNA-21 inhibits Smad7 expression to promote TGF-β/Smad signaling-mediated liver fibrosis, and chlorogenic acid inhibits miRNA-21 expression to suppress SSLF (Wang Y. et al., 2017; Cui et al., 2021). miR-130a-3p inhibits the expression of the TGF-β receptor, which suppresses the TGF-β signaling-mediated expression of fibrosis-related genes (Liu et al., 2021a). miR-200a can negatively regulate SSLF, which could be related to the downregulation of TGF-β2 levels, but the target genes of miRNA-200a in SSLFs are unclear (Xu et al., 2021). TLR2 signaling plays an important role in SSLF development, and overexpression of miR-92a-2-5p could block TLR2 signaling (Zhao et al., 2019). miRNAs are widely involved in the post-transcriptional regulation of genes and provide interesting protein molecules for researching SSLF (**Table 1**).

**Table 1:** The role of MicroRNA in schistosoma-associated liver fibrosis.

MicroRNA	Sources	Effects	Reference
miRNA-33	schistosoma	Enhanced stability of TGF-β receptor mRNA	Wang et al., 2022
miRNA-71a	schistosoma	Targeting Sema4D to inhibit liver fibrosis	Wang L. et al., 2020
miRNA-1	schistosoma	Targeting SFRP1 in HSCs and upregulating Wnt/β-catenin signaling to promote SSLF	Zhang H. et al., 2019
MiRNA-182	Hosts	Targeted inhibition of FOXO1 expression promotes the activation and proliferation of HSCs	Huang et al., 2018
Let-7b	Hosts	Targeting type I collagen to inhibit collagen deposition	Tang et al., 2017; Sun X. et al., 2021
miRNA-203-3p	Hosts	Inhibition of IL-33 expression	He et al., 2018b
miRNA-27b	Hosts	Targeting KSRP to inhibit the degradation of TGF-β mRNA	Sundaram et al., 2013
miRNA-351	Hosts	Reduction of VDR signal	He et al., 2018a
MiRNA-21	Hosts	Inhibition of Smad7 expression to promote TGF-β/Smad signaling	Wang Y. et al., 2017; Cui et al., 2021
miR-130a-3p	Hosts	Inhibition of TGF-β receptor expression	Liu L. et al., 2021
miR-92a-2-5p	Hosts	Blocking the TLR2 signaling pathway	Zhao et al., 2019

Sema4D, semaphorin 4D; SFRP1, Secreted frizzed-related protein 1; FOXO1, Forkhead box other 1; KSRP, KH-type splicing regulatory protein; VDR, Vitamin D receptor; TLR2, Toll-like receptor2.

## NLRP3 inflammasome and TLR2/4 signaling pathway

### NLRP3 inflammasome

NLRP3 is a member of the NOD-like receptors (NLRs) family, which is a multiprotein complex that plays an important role in SSLF (Lu et al., 2017; Mangan et al., 2018). The NLRP3 inflammasome consists of NLRP3, adaptor protein apoptosis-associated speck-like protein (ASC), and pro-caspase-1, which is activated by various agonists (Meng et al., 2016; Zhang W. J. et al., 2019). The three main molecular mechanisms of NLRP3 activation are ROS production, increased lysosomal membrane permeability, potassium efflux, and caspase-1 shearing of precursors of IL-1β and IL-18 to promote their activation (Meng et al., 2016; Lu et al., 2017; Zhang W. J. et al., 2019; Zhen and Zhang, 2019). IL-1β promotes the activation and proliferation of HSCs and recruits high levels of inflammatory cytokines, and also induces the activation of T cells and macrophages to promote worm egg granulomas and liver fibrosis (Palacios-Macapagal et al., 2017; Liu et al., 2019).

**Figure 2:**
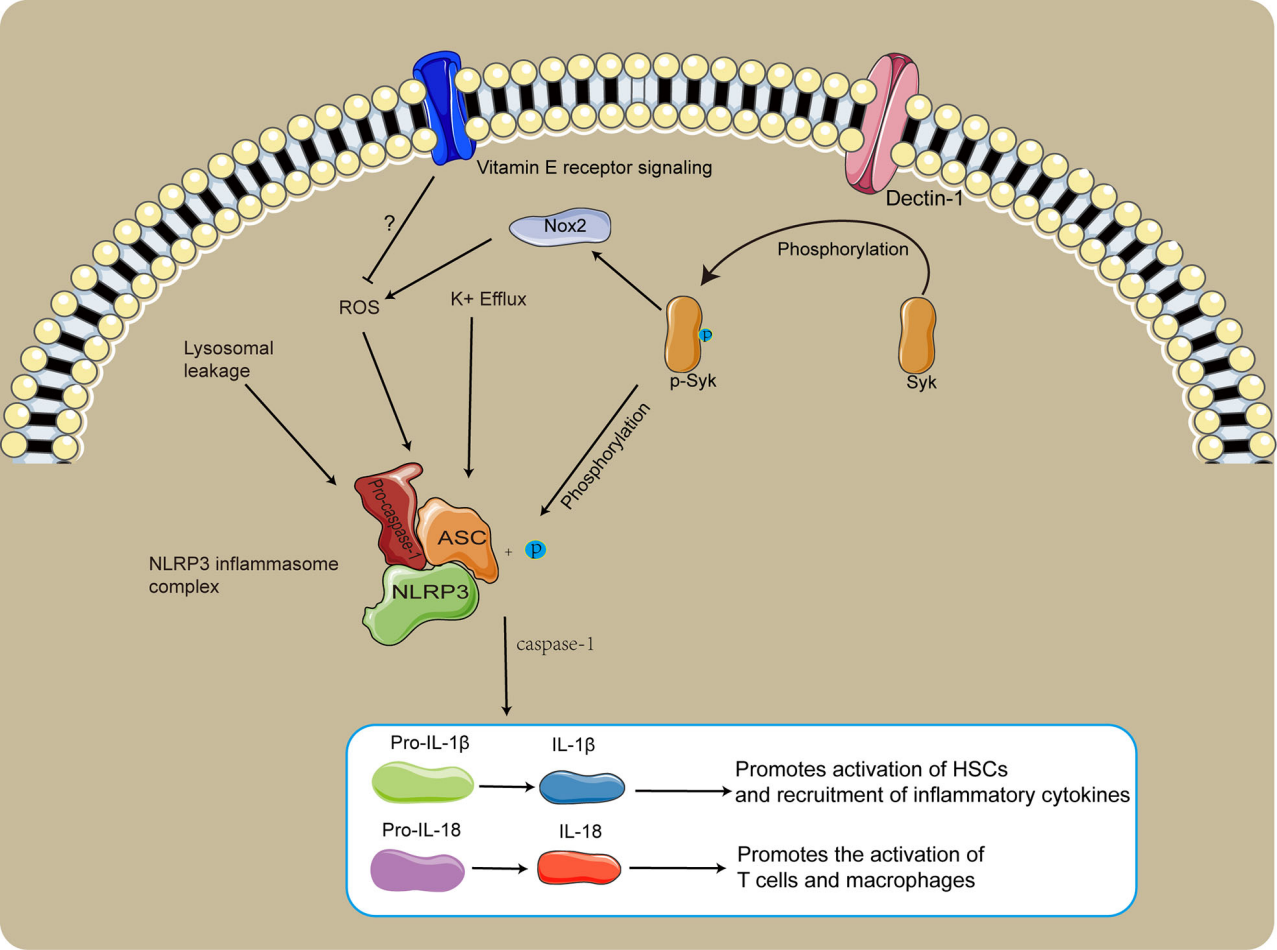
The role of NLRP3 in SSLF. The Dectin-1/Syk signaling pathway promotes NLRP3 inflammasome activation *via* phosphorylation of ASC. p-syk dependent NOX2 induced production of ROS facilitates NLRP3 inflammasome activation. Vitamin E likely inhibits SSLF by suppressing ROS production and relieving inflammatory injury.

There are high levels of IL-1β and IL-18 in the liver of schistosome-infected mice, and in HSC cells with highly activated NLRP3 inflammasome (Meng et al., 2016). Dendritic cell-associated C-type lectin-1 (Dectin-1) is a C-type lectin-like pattern receptor that recruits and phosphorylates spleen tyrosine kinase (Syk) (Sun W. et al., 2021). Activation of NLRP3 in HSCs caused by schistosome infections depends on the Dectin-1/Syk signaling pathway (**Figure 2**), while inhibition of NLRP3 inflammasome activation significantly reduces egg granulomas and liver fibrosis (Lu et al., 2017). Phosphorylated syk(p-syk) promotes activation of the NLRP3 inflammasome by phosphorylated ASC, and promotes NADPH oxidase2 (NOX2) signaling producing ROS to indirectly activate the NLRP3 inflammasome (**Figure 2** **) (** Lu et al., 2017; Yu et al., 2021). Induction of caspase-1-dependent apoptosis by ROS mediates extensive inflammatory damage in schistosome-infected mice, and ROS blockades inhibit inflammation and promote M1-type polarization of macrophages (Kong et al., 2019). Oxidative stress promotes the dissociation of thioredoxin-interacting protein (TXNIP) from thioredoxin and TXNIP can activate the NLRP3 inflammasome, but taurine inhibits inflammation by inhibiting the TXNIP/NLRP3 signaling pathway (Liu et al., 2019). Treatment of schistosome-infected mice with vitamin E could reduce liver fibrosis and egg granulomas (Wang et al., 2012). Vitamin E could be involved in regulating SSLF by inhibiting ROS production and relieving inflammatory damage, though its regulatory mechanism is unclear (**Figure 2**).

High activation of NLRP3 in the liver of schistosome-infected mice promoted extensive tissue damage and inflammatory responses, which induced connective tissue proliferation promoting SSLF development (Meng et al., 2016; Carson et al., 2018; Zhang W. J. et al., 2019). Inhibiting excessive activation of the inflammatory response and tissue damage by inhibiting NLRP3 signaling has a positive significance for SSLF therapy.

### TLR2/4 signaling pathway

The toll-like receptor (TLR) is a pattern recognition receptor that senses endogenous and exogenous dangerous molecules to produce high levels of pro-inflammatory cytokines, which activate lymphocytes to generate adaptive immune responses and destroy invading pathogens or damaged cells (Lim and Staudt, 2013; Chen L. et al., 2021). The TLR family consists of 13 molecules that are widely expressed on immune and non-immune cells in mice. Of these, TLR2/4 is the most important receptor for the recognition of schistosome components (Zhang et al., 2011; Liu et al., 2020). However, TLR2 and TLR4 have different effects on schistosome infection. TLR2 deficiency reduces egg burden and promotes T cell activation, and TLR4 deficiency aggravates schistosome immunopathology and liver fibrosis (Zhang et al., 2011; Liu et al., 2020). TLR2 deficiency upregulates IFN-γ and IL-4, which could be related to TLR2 signaling-induced regulatory T cell differentiation (Layland et al., 2007; Burton et al., 2010). TLR2 signaling in dendritic cells promotes PD-L2 expression to maintain immunosuppression (Gao et al., 2013). TLR2 signaling induced by SEA stimulation promotes M2 polarization in macrophages, which is beneficial for developing liver fibrosis (Gong et al., 2018). TLR2 signaling can contribute to maintaining the immunosuppressive state of the schistosome-infected microenvironment and suppressing T cell activation (Zhang et al., 2011). TLR4 signaling promotes HSC activation and ECM deposition by activating the COX2/PGE2 axis to upregulate Prostaglandin E2 (**PGE2**) expression (Chen L. et al., 2021). TLR4 signaling promotes M1-type polarization of macrophages, which has the opposite effect of TLR2 (Xu et al., 2014). TLR2/4 plays an important role in regulating the Th1/Th2 balance, which is highly correlated with the development of SLLF. However, the role of TLR2/4 in schistosome infections requires further study.

## TGF-*β*/Smad axis and IL-13 signing promote SSLF

### TGF-β/Smad axis

There are three subtypes of TGF-β with partially overlapping functions. In liver fibrosis, the most extensively researched is TGF-β1, which is the main pro-fibrotic cytokine driving HSC activation and proliferation (Dewidar et al., 2019). Chronic liver injuries induced by schistosome infections provoke a continuous inflammatory response, and liver sinusoidal endothelial cells (LSECs) are the first cell type to be affected after liver injuries (Parola and Pinzani, 2019; Ruart et al., 2019). Chronic inflammation is maintained by the increased production of reactive oxygen radicals (ROS) and other oxidative stress products (Ruart et al., 2019). Kupffer cells also play an important role in sensing liver injuries and triggering inflammation. With the continued depletion of Kupffer cells, the liver recruits mononuclear-derived macrophages to maintain chronic inflammation and liver fibrosis (Tacke and Zimmermann, 2014; Wang et al., 2018). Persistent liver injuries and chronic inflammatory responses mediated by schistosome infections are important factors in the development of liver fibrosis. The type II immune microenvironment created by schistosome infections promotes TGF-β1 secretion by macrophages, hepatic stellate cells, and hepatocytes in the liver (Li Y. Q. et al., 2017; Wang S. et al., 2020). Inflammatory and injurious stimulants induce high levels of TGF-β, and TGF-β binding to TGF-β receptor complexes promotes SMAD2/SMAD3 phosphorylation (Dewidar et al., 2019). Phosphorylated Smad2/Smad3 combines with Smad4 to form a protein complex, and the Smad complex performs nuclear translocation to promote transcription of target genes, with the expression of many fibrosis-related genes dependent on Smad3 (Chen B. L. et al., 2013; Chen et al., 2019b; Wang et al., 2022). SMAD3 has a low affinity for DNA; therefore, the Smad2/3/4 protein complex recruits transcriptional co-activators to stabilize the activation complex (Dewidar et al., 2019). Smad protein complexes promote collagen deposition by enhancing the expression of liver fibrosis-related genes and matrix metalloproteinase inhibitors (He et al., 2018a). Phosphorylated Smad1/5/8 competes with phosphorylated Smad2/3 to bind Smad4 to suppress the transcription of liver fibrosis-related genes, and BMP-7 promotes the phosphorylation of Smad1/5/8 in hepatic stellate cells (Chen et al., 2019b). Intense TGF-β signaling induces excessive inflammatory responses and collagen deposition, while also activating the negative feedback regulation of TGF-β signaling by Smad7 (Yang et al., 2020). Smad7 is an interesting target for anti-SSLF therapy, and Smad7 overexpression in HSCs significantly relieves liver fibrosis (Dooley et al., 2008). Sedum sarmentosum Total Flavonoids (SSTF) have anti-inflammatory and antioxidant effects, which induce high expression of Smad7 in HSCs to inhibit TGF-β signaling in Schistosoma infection (Yang et al., 2020). However, the mechanism behind SSTF-induced high expression of Smad7 has not yet been researched. Previous studies have demonstrated that Sema4Dis constitutively expressed on T cells and is involved in regulating T cell activation (Kuklina et al., 2017). However, schistosome infections induce HSCs highly expressing Sema4D and Sema4D receptors, and Sema4D signaling enhances TGF-β signaling, while inhibition of Sema4D significantly reduces SSLF (Wang L. et al., 2020). As a natural flavonoid with anti-inflammatory and antioxidant effects, myricetin can inhibit TGF-β1/smad signaling in HSCs to inhibit SSLF (Huang et al., 2020). TGF-β1/Samd signaling promotes increased expression of CTGF, which promotes HSC activation and ECM deposition (Kamdem et al., 2018). TGF-β has various biological functions, and the inhibition of TGF-β signaling has a positive effect during antifibrotic therapy for schistosomiasis (Dewidar et al., 2019; Larson et al., 2020). Current research indicates that TGF-β/Smad is an important pro-fibrotic signal in schistosome infections. The targeted regulation of TGF-β/Smad signaling could provide a breakthrough for antifibrotic therapy of schistosomiasis (**Table 2**).

**Table 2:** The role of cytokines in schistosoma-associated liver fibrosis.

Cytokines	Sources	Receptor	Effects	Reference
IL-17	γδT cells, Th17, NKT	IL-17 Receptor	Induced Inflammation and migration of neutrophils	Webster et al., 2014; Zheng et al., 2017; Sun et al., 2020
IL-33	cell nucleus	ST-2	Induced of macrophage M2 polarization and recruitment of ILC2s	Li et al., 2019; He et al., 2018b
TGF-β	Macrophages, hepatic stellate cells and hepatocytes	TGF-β Receptor	Promote activation of HSCs and expression of fibrosis-related genes	Li Y. Q. et al., 2017; Wang S. et al., 2020; Chen B. L. et al., 2013
IL-13	Hepatic parenchymal and immune cells	IL-4α, IL-13Rα, IL13Rα2	Induced expression of fibrosis-associated genes in HSCs	Chiaramonte et al., 2003; Figueiredo et al., 2016

ILC2s, Group 2 innate lymphoid cells; HSCs, hepatic stellate cells.

## IL-13

Both parenchymal and immune cells can produce IL-13 in the liver of schistosome-infected mice, and IL-13 signaling induces the expression of type I collagen and other fibrosis-related genes in HSCs (Liu et al., 2012; Zheng et al., 2015). The IL-13 signaling receptor complex consists of IL-4Rα, IL-13Rα1, and IL13Rα2 (Figueiredo et al., 2016). IL-13Rα1 combines with IL-4Rα1 to constitute a functional receptor for IL-13 signaling, and IL-13 Rα2 is a bait receptor capable of blocking IL-13 signaling (Rahaman et al., 2002; Zheng et al., 2015). However, schistosome infections induced high levels of IL-13 Rα2 expression by macrophages, which are positively correlated with the development of liver fibrosis (Wang et al., 2009). Gadolinium chloride (GdCl_3_) relieves SSLF by scavenging macrophages and decreasing IL13Rα2 levels in the liver, which is likely related to the reduction of TGF-β production by macrophages induced by IL13Rα2 (Zheng et al., 2015). Macrophage IL-13Rα2 inhibits Matrix metalloproteinase 13(MMP-13) expression and thus ECM degradation, and Matrix metalloproteinase 12(MMP-12) deficient inhibition of IL-13Rα2 expression recovers the activity of MMP-13 (Madala et al., 2010). Specific knockdown of IL-13 Rα2 in macrophages could result in important discoveries relevant to SSLF research, however, none have yet been reported.

IL-13 combines with the IL-13 functional receptor to promote JAK/STAT6 phosphorylation, and phosphorylated STAT6 to constitute homodimers and nuclear translocations to induce the expression of fibrosis-related genes (Du et al., 2016; Li et al., 2016; Wang L. et al., 2020). IL-13 activates the ERK-MAPK signaling pathway to promote Smad1/2 phosphorylation and increase CTGF expression to induce HSC activation; however, this must be confirmed in SSLF (Liu et al., 2011; Wang Y. et al., 2017). Many antifibrotic drugs inhibit IL-13 signaling to inhibit the progression of SSLF, such as paeoniflorin, corilagin, and chlorogenic acid (Li et al., 2010; Li et al., 2016; Wang Y. et al., 2017). C/EBP Homologous Protein (CHOP) is an endoplasmic reticulum stress-induced transcriptional regulator, which inhibits the IL-13/STAT6 signaling pathway to suppress M2 macrophage polarization and SSLF (Duan et al., 2019). Sema4D combined with its receptor upregulates IL-13 signaling to promote SSLF, and targeted inhibition of Sema4D significantly inhibits SSLF (Wang L. et al., 2020). IL-13 signaling plays an important role in the development of SSLF (**Table 2**). However, numerous cell types secrete IL-13 in the liver. Therefore, we recommend analyzing total IL-13 levels in the liver or researching IL-13 downstream signaling regulation in specific cell types.

## IL-17 and IL-33 promote SSLF

### IL-17

IL-17 is a pro-inflammatory cytokine involved in many inflammatory and infectious diseases, including schistosomiasis, and blocking IL-17 significantly reduces egg granuloma and liver fibrosis (Zhang et al., 2015). T Helper 17 (Th17) cells, natural killer T (NKT) cells, and gamma delta (**γδ**) T cells are important sources of IL-17 (Webster et al., 2014; Sun et al., 2020). In the schistosome-infected liver, γδ T cells account for 1-2% of all T lymphocytes, however, the most important source of IL-17 is γδ T cells (Chen D. et al., 2013). γδ T cell deficiency reduces IL-17 levels in the liver, and IL-17 levels tend to be positively correlated with the progression of SSLF (Sun et al., 2020). The γδ T cells in the schistosome-infected liver are mainly composed of Vγ1 and Vγ2, with Vγ1 γδ T cells secreting IFN-γ and Vγ2 γδ T cells secreting both IFN-γ and IL-17A (Zheng et al., 2017). IL-17 receptors are highly expressed on the surface of neutrophils, while IL-17 secreted by Vγ2 γδ T cells recruited neutrophils to migrate to the area of egg granulomas and TGF-β secreted by neutrophils promotes the progression of SSLF (Zheng et al., 2017; Sun et al., 2020). IL-17 induces Type I collagen gene expression in HSC cells by phosphorylating STAT3 to promote liver fiber (Zhou et al., 2019). ApoE-deficient mice have a severe lipid metabolism disorder and hyperactivated inflammatory response. High levels of IL-17 in the liver of schistosome-infected ApoE-deficient mice aggravate egg granulomas and fibrosis (Guan et al., 2020). The role of IL-17 in promoting schistosome immunopathology does not need to be emphasized; however, the mechanism of IL-17 regulation of SSLF requires further research (**Table 2**).

### IL-33

IL-33 is a part of the IL-1 family and is normally present in the nucleus and released into the extracellular compartment in response to tissue damage (Bai et al., 2021). IL-33 plays an important role in immunity to schistosome infection, and IL-33 levels are positively correlated with the progression of egg granuloma and liver fibrosis (Zhang et al., 2021). IL-33 induces macrophage M2-type polarization and releases IL-5 and IL-13 to promote liver fibrosis (Li et al., 2019). IL-33 promotes the recruitment of Group 2 innate lymphoid cells (ILC2s) in the liver, and secretion of IL-13 by ILC2s promotes the development of liver fibrosis (He et al., 2018b). IL-33 contributes to the maintenance of type II immune microenvironments in the liver of schistosome-infected mice. During chronic infections, Hepatic progenitor cells (HPCs) inhibit the secretion of IL-33 to promote tissue restoration and inhibit the development of liver fibrosis (Zhang et al., 2021). Tissue Transglutaminase (tTG) regulates cell death and cytoskeletal rearrangement, however, tTG is also involved in the regulation of SSLF. The tTG upregulation of IL-33 promotes liver fibrosis and worm egg granuloma (Tang et al., 2015; Li et al., 2019). ST2 is a unique IL-33 receptor, and appropriate inhibition of IL-33/ST2 reduces the immunopathology of schistosomes; however, knockdown of IL-33 or ST2 exacerbates schistosomiasis liver pathology (Li et al., 2019; Bai et al., 2021). IL-33 deficiency could activate excessive Th17 immune response. The IL-33/ST2 axis is influenced by multiple immune cells and cytokines during immunity to Schistosoma infection. There could be additional receptors for IL-33 besides ST2. Therefore, we recommend exploring additional receptors for IL-33 and lateral regulation of the IL-33/ST2 axis (**Table 2**).

## The role of HMGB1 in SSLF

High mobility group box-1 protein (HMGB1) is a highly conserved nuclear DNA binding protein that plays a regulatory role in gene transcription as a structural cofactor (Chen et al., 2004). HMGB1 can be released from dead cells or secreted from intrinsic immune cells, and HMGB1 promotes the secretion of inflammatory cytokines (Rauvala and Rouhiainen, 2007). HMGB1 promotes the proliferation and activation of HSCs to promote liver fibrosis during SSLF progression, and high levels of HMGB1 at the injury site recruit fibroblasts, endothelial cells, and smooth muscle cells to promote tissue injury repair (Vicentino et al., 2018; Chen H. et al., 2021). However, this decompensation repair has resulted in liver fibrosis due to persistent inflammation and injuries, and HMGB1 promotes the migration of immune cells to the area of egg granulomas (Vicentino et al., 2018). HMGB1 inhibitors significantly inhibit SSLF, which indicates that HMGB1 plays a role in promoting liver fibrosis, though its mechanism is unknown (Vicentino et al., 2018; Chen H. et al., 2021). HMGB1 is derived from passive leakage from dead cells and primary release from intrinsic immune cells, and the primary source and mechanism of HMGB1 in SSLF must be clarified. In conclusion, high levels of HMGB1 were positively correlated with SLLF, HMGB1 was extensively post-translationally modified, and the role of HMGB1 in SSLF must be further investigated (Chen et al., 2004).

## NK cells and NKT cells

NK cells are natural immune cells that directly lyse or induce apoptosis of target cells by secreting granzyme, perforin, and FasL/Fas pathways (Liu et al., 2021b). The natural killer group 2, member D(NKG2D), is an essential NK cell activation and function-related molecule, and retinoic acid early induction 1 (RAE 1) is one of the ligands of NKG2D (Hou et al., 2012a **).** At 2 to 4 weeks of schistosome infection, HSC cells are highly expressive of RAE 1 and tumor necrosis factor-related apoptosis-inducing ligand (TRAIL), which facilitate NK cell recognition and the killing of HSCs (Hou et al., 2012a; Hu et al., 2020). Murine UL16-binding protein-like transcript 1 (MULT1) is the strongest ligand for NKG2D, and overexpression of MULT1 enhances NK cell activity to inhibit SSLF (Yang et al., 2022). NK cells express a variety of TLRs, and schistosome infection induces high TLR3 expression in NK cells (Qu et al., 2018 **).** Polyinosinic-polycytidylic acid (poly I:C)-TLR3 signaling post-translationally promotes NKG2D expression and IFN-g secretion in NK cells (Hou et al., 2012b). NK cells secrete IFN-γ to induce HSC apoptosis and block the cell cycle, and the absence of NK cells promotes SSLF (Hou et al., 2012a). In general, IFN-g secretion by NK cells and direct killing of HSCs inhibited SSLF in the early stages of schistosome infections. However, with schistosome egg production, the number of NK cells in the liver increased but their activities were dramatically inhibited (Hu et al., 2020). Therefore, it is important to identify how to restore NK cell activity to resist SSLF.

NKT is an innate immune cell subset that expresses both NK and T cell markers but is essentially a specialized T cell subset (Faveeuw et al., 2008). NKT cells can be classified as either invariant NKT(iNKT) or non-invariant NKT(non-iNKT) based on the variability of their TCR (Noma et al., 2015). CD1d is a highly conserved MHC class I molecule, and NKT cell recognition of APC-presented antigenic peptides depends on CD1d (Nishioka et al., 2018). Antigen-peptide processing by antigen-presenting cells stimulates NKT cells to produce high levels of IFN-γ and IL-4; however, this conflicting effect is undertaken by different NKT subsets (Lei et al., 2021). iNKT cells promote upregulated levels of IFN-γ, while non-iNKT cells promote upregulated levels of IL-4, which indicates that these two cell types could play opposite roles in the development of SSLF (Mallevaey et al., 2007). Schistosoma haematobium infections lead to increased numbers of NKT cells in the liver and decreased CD1d expression in hepatocytes, which could be associated with CD1d-mediated apoptosis of NKT cells (Lei et al., 2021). IL-30 promotes the recruitment of NKT cells in the liver and upregulates the expression of NKG2D, promoting the killing of HSCs by NKT cells (Mitra et al., 2014). However, the role of IL-30 in SSLF has yet to be validated. The major cell subsets of NKT cells could play opposite roles in SSLF, and research targeting NKT cell subsets could lead to breakthroughs for SSLF therapy.

## Conclusion

Liver fibrosis caused by schistosomiasis is still a problem when treating schistosomiasis, and anti-fibrosis therapy are important for improving the quality of life of patients and restoring their liver function. The main reasons for liver fibrosis are continuous liver injury and inflammatory stimulation. The most important difference between SSLF and other types of liver fibrosis is the persistent damage and inflammatory response mediated by eggs and SEA, which is unique to SSLF. SEA inhibits the proliferation and activation of HSCs, and schistosome-derived miRNAs are involved in regulating SSLF. The levels of expression of some critical pro-fibrotic cytokines could be affected by schistosomes. The schistosome infection microenvironment is composed of extremely complex components that could play opposing roles in the progression of liver fibrosis. Therefore, we must understand the functions and mechanisms of the molecules involved in regulating SSLF. This paper reviews the mechanisms of the major molecules or signaling pathways involved in the development of SSLF and discusses potential future research topics.

## Author contributions

ZL and LZ drafted and revised the manuscript, and LL and YL reviewed and revised the manuscript for publication. All authors contributed to the article and approved the submitted version.

## Funding

This work was supported by the National Natural Science Foundation of China (32070898 to YL, 81471595 to YL, 32170879 to LZ, 81601360 to LZ, 81501342 to LL, and U1904157 to LL).

## Conflict of interest

The authors declare that the research was conducted in the absence of any commercial or financial relationships that could be construed as a potential conflict of interest.

## Publisher’s note

All claims expressed in this article are solely those of the authors and do not necessarily represent those of their affiliated organizations, or those of the publisher, the editors and the reviewers. Any product that may be evaluated in this article, or claim that may be made by its manufacturer, is not guaranteed or endorsed by the publisher.
